# Supplementary material for: Bacterial Community with Plant Growth-Promoting Potential Associated to Pioneer Plants from an Active Mexican Volcanic Complex
Source: Microorganisms. 2022 Aug 4;10(8):1568. doi: 10.3390/microorganisms10081568 (PMC9413462; doi:10.3390/microorganisms10081568)
Supplement: Supplementary file 1 [file microorganisms-10-01568-s001.zip › microorganisms-1841673-SI.pdf]

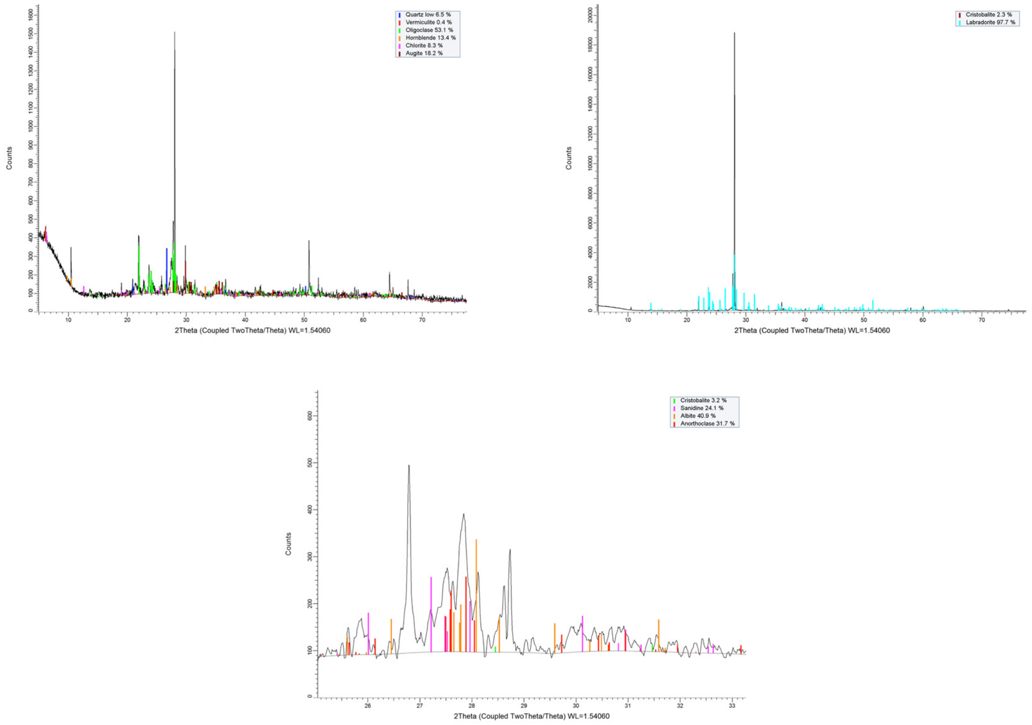

Figure S1. Peaks of the minerals detected in *C. aemula* rhizosphere samples by X-ray diffraction (XRD) analysis.

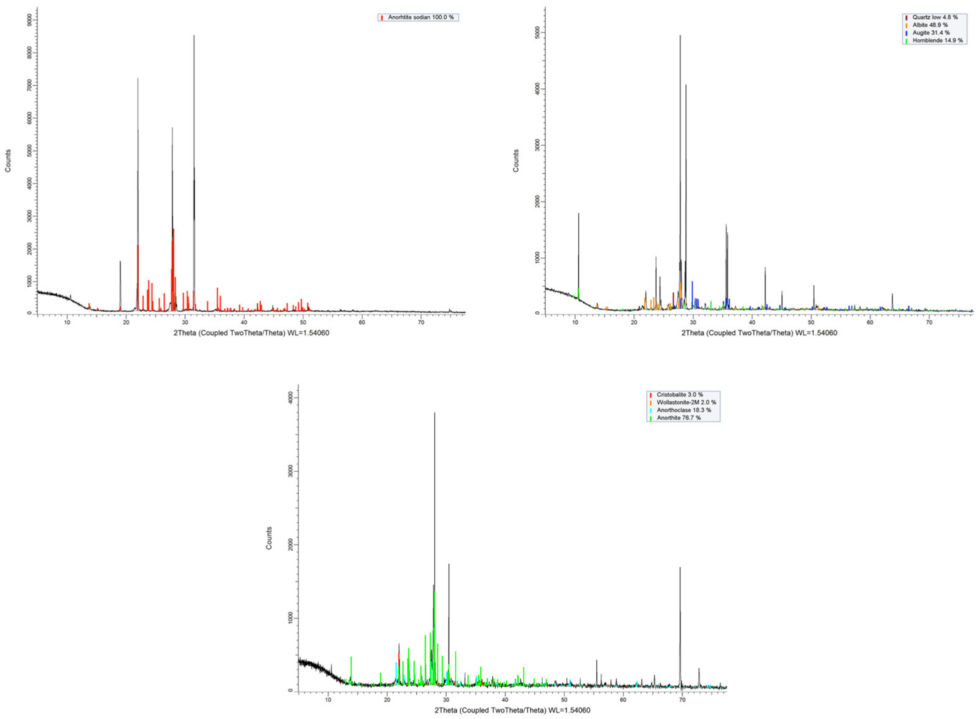

Figure S2. Peaks of the minerals detected in *A. glomeratus* rhizosphere samples by X-ray diffraction (XRD) analysis.

Table S1. Minerals detected in the rhizospheric soil of the pioneer plants from El Chichón volcano crater-lake

Mineral	Rhizosphere *Cheilanthes aemula*	Rhizosphere *Andropogon glomeratus*
Aerinite	f	f
Alabandite	*	f
Albite	**	***
Andesine	f	f
Apuanite	f	f
Arsenolite	*	f
Berlinite	*	f
Blossite	f	f
Calcite	f	*
Canfieldite	f	f
Chkalovite	f	f
Cristobalite	***	***
Eglestonite	**	**
Eliseevite	f	f
Falsterite	f	*
Ferrarisite	f	f
Graphite	*	f
Iriginite	f	f
Intersilite	f	f

Koechlinite	f	f
Labradorite	**	***
Lintisite	f	f
Maricopaite	f	f
Melanovanadite	*	*
Metahewettite	*	*
Muscovite	*	*
Quartz	**	**
Sassolite	f	f
Stenhuggarite	f	f
Tazheranite	*	f
Walfordite	f	f
Yeelimite	f	f

The intensities of X-ray peaks in the XRD analysis are shown as: *** intense; ** weak; * minor; f: faint and blank none.

Table S2. Concentration of metals in the rhizospheric soil of the pioneer plants from El Chichón volcano crater-lake

Metals	Metal concentration (mg kg^{-1}) in rhizosphere		*p*-value [b]
	Cheilanthes aemula	*Andropogon glomeratus*	
Al	5780.67 ± (635.7)[a]	3270.33 ± (367.1)	0.00407*
As	0.3 ± (0.0)	0.73 ± (0.05)	0.00020*
Ba	56.8 ± (4.94)	88.1 ± (9.02)	0.00621*
Ca	6410.67 ± (333.3)	4631.67 ± (702.36)	0.01663*
Cd	0.066 ± (0.05)	0.26± (0.05)	0.01323*
Co	1.93 ± (0.15)	0.86 ± (0.11)	0.00064*
Cr	3.6 ± (0.36)	1.13 ± (0.15)	0.00040*
Cu	31.3 ± (3.57)	31.6 ± (1.99)	0.90523*
Fe	8965.33 ± (981.8)	1289.0 ± (131.93)	0.00017*
K	1301.0 ± (214.0)	528.7 ± (30.99)	0.00347*
Mg	3398.0 ± (311.3)	415.23 ± (32.10)	0.00007*
Mn	123.46 ± (12.71)	34.26 ± (4.68)	0.00033*
Na	540.43 ± (39.01)	572.66 ± (32.97)	0.33584*
Ni	1.2 ± (0.17)	3.0 ± (3.46)	0.42024*
Pb	7.53 ± (0.70)	3.63 ± (0.80)	0.00322*
Sr	99.4 ± (10.49)	87.63 ± (9.62)	0.22555*
V	61.96 ± (6.75)	19.96 ± (1.98)	0.00049*
Zn	21.06 ± (1.95)	11.26 ± (1.11)	0.00164*

[a] Mean values of three replicates. The values in parenthesis are standard deviation; [b] p-value according to Student's t-test ($p < 0.05$). * Significant difference between *A. glomeratus* and *C. aemula* plants
